# Diagnostic accuracy of artificial intelligence for detecting gastrointestinal luminal pathologies: A systematic review and meta-analysis

**DOI:** 10.3389/fmed.2022.1018937

**Published:** 2022-11-04

**Authors:** Om Parkash, Asra Tus Saleha Siddiqui, Uswa Jiwani, Fahad Rind, Zahra Ali Padhani, Arjumand Rizvi, Zahra Hoodbhoy, Jai K. Das

**Affiliations:** ^1^Department of Medicine, Aga Khan University, Karachi, Pakistan; ^2^Center of Excellence in Women and Child Health, Aga Khan University, Karachi, Pakistan; ^3^Head and Neck Oncology, The Ohio State University, Columbus, OH, United States; ^4^Institute for Global Health and Development, Aga Khan University, Karachi, Pakistan; ^5^Department of Pediatrics and Child Health, Aga Khan University, Karachi, Pakistan

**Keywords:** artificial intelligence, systematic review, gastroenterology, diagnostic accuracy, pathologies

## Abstract

**Background:**

Artificial Intelligence (AI) holds considerable promise for diagnostics in the field of gastroenterology. This systematic review and meta-analysis aims to assess the diagnostic accuracy of AI models compared with the gold standard of experts and histopathology for the diagnosis of various gastrointestinal (GI) luminal pathologies including polyps, neoplasms, and inflammatory bowel disease.

**Methods:**

We searched PubMed, CINAHL, Wiley Cochrane Library, and Web of Science electronic databases to identify studies assessing the diagnostic performance of AI models for GI luminal pathologies. We extracted binary diagnostic accuracy data and constructed contingency tables to derive the outcomes of interest: sensitivity and specificity. We performed a meta-analysis and hierarchical summary receiver operating characteristic curves (HSROC). The risk of bias was assessed using Quality Assessment for Diagnostic Accuracy Studies-2 (QUADAS-2) tool. Subgroup analyses were conducted based on the type of GI luminal disease, AI model, reference standard, and type of data used for analysis. This study is registered with PROSPERO (CRD42021288360).

**Findings:**

We included 73 studies, of which 31 were externally validated and provided sufficient information for inclusion in the meta-analysis. The overall sensitivity of AI for detecting GI luminal pathologies was 91.9% (95% CI: 89.0–94.1) and specificity was 91.7% (95% CI: 87.4–94.7). Deep learning models (sensitivity: 89.8%, specificity: 91.9%) and ensemble methods (sensitivity: 95.4%, specificity: 90.9%) were the most commonly used models in the included studies. Majority of studies (*n* = 56, 76.7%) had a high risk of selection bias while 74% (*n* = 54) studies were low risk on reference standard and 67% (*n* = 49) were low risk for flow and timing bias.

**Interpretation:**

The review suggests high sensitivity and specificity of AI models for the detection of GI luminal pathologies. There is a need for large, multi-center trials in both high income countries and low- and middle- income countries to assess the performance of these AI models in real clinical settings and its impact on diagnosis and prognosis.

**Systematic review registration:**

[https://www.crd.york.ac.uk/prospero/display_record.php?RecordID=288360], identifier [CRD42021288360].

## Introduction

Gastrointestinal (GI) pathologies contribute to a significant burden of disease worldwide. With 89 million global disability-adjusted life years (DALYs), GI pathologies contributed 3.5% (2,280 million cases) to the total global DALYs in 2019, with a greater prevalence in low and middle-income countries (LMICs) (1). In 2018, there were more than 36⋅8 million ambulatory visits in the United States for GI symptoms and 43⋅4 million had a primary GI diagnosis. Annually, a total of 22.2 million GI endoscopies were performed, with 284,844 new GI cancers diagnosed and 255,407 deaths (2). Other parts of the world, including LMICs also have an increasing burden of GI pathologies, as 80% of the esophageal cancer burden of the world is from LMICs, with poor survival (3). The morbidity and mortality due to GI causes is higher than other common pathologies and hence underscores a significant burden that GI adds to the overall health care system.

Despite this high burden of disease, there are multiple challenges that hinder the provision of optimal GI care. In LMICs, it is often the lack of resources such as endoscopy equipment and availability of skills and experts for timely diagnosis and intervention (4). In high income regions, these challenges include high costs along with discrepancies in facilities and training (5). Overcoming these challenges would require significant amount of resources and time. Although progress has been made globally to enhance these skills, this capacity is still lagging. However, innovations in technology have proven to be a beacon to overcome these challenges adeptly and efficiently.

The introduction of artificial intelligence (AI) in health care has led to innovations in diagnosis, management and prognosis of many conditions at a fast pace. AI algorithms in gastroenterology have been studied over many years to automate the interpretation of diagnostic procedures in gastroenterology albeit with varying levels of success. Since 2010, AI has explored multiple procedures and pathologies in gastroenterology (6). The AI models have been applied to the interpretation of endoscopy, pill video endoscopy, ultrasound manometry, and microcytoscopy (7–17). Traditionally, these procedures yield large amounts of data which require an expert’s time and attention to draw clinical conclusions. However, AI models in these studies have shown to recognize polyps, areas of inflammation, and degrees of inflammation accurately. A recent randomized controlled trial reported much lower miss rates for a deep learning model for polyp detection compared to the standard (20.1% vs. 31.2%) (18). The massive influx and availability of data, along with promising performance of AI in lesion detection, makes incorporation of AI in healthcare promising. However, there is a need to synthesize the existing literature to quantify the accuracy of AI algorithm in detection of GI disease.

The primary objective of this systematic review and meta-analysis was to assess the diagnostic accuracy of AI models compared with the gold standard of experts and histopathology for the diagnosis of various gastrointestinal luminal pathologies, including polyps, neoplasms, inflammatory bowel disease (IBD), celiac disease, and Barrett’s esophagus. The secondary objective was to describe the diagnostic accuracy of different types of AI models for the diagnosis of each GI luminal pathology.

## Methods

The protocol for this review was prospectively registered at PROSPERO (CRD42021288360). We followed the Preferred Reporting Items for Systematic reviews and Meta-Analyses (PRISMA) guidelines for diagnostic test accuracy for analysis reporting in this publication (19).

### Eligibility criteria and search strategy

We included all observational studies that reported the diagnostic results of an AI algorithm for the detection of GI luminal pathologies when compared to a reference standard (expert opinion or consensus, histopathology, or laboratory testing such as urea breath test for H. pylori etc.). No restrictions were applied based on the age at diagnosis or type of AI algorithm used in the study. Studies with an unclear description of reference standard or type of GI luminal pathologies, published in a language other than English, and those that graded the severity of an already diagnosed disease were excluded. We excluded letters, opinions, preprints, scientific reports, and narrative reviews. Studies based on animals or non-human samples or that presented duplicate data were excluded.

We searched PubMed, CINAHL, Wiley Cochrane Library, and Web of Science electronic databases to identify relevant articles published until January 27, 2021. The keywords used for the search included, “Algorithms,” “Artificial Intelligence,” “Machine Learning,” “Deep Learning,” “Supervised Machine Learning,” “Unsupervised Machine Learning,” “Gastroenterology,” “Celiac Disease,” “Inflammatory Bowel Disease*,” “Irritable Bowel Syndrome,” “Polyp*,” “Crohn Disease,” “Gastro*,” “Endoscopy,” “Scopy,” “Capsule Endoscopy,” “Endomicroscopy,” “Colonoscopy,” “Ultrasound Manometry,” “Diagnosis,” “Diagnos*,” “Accuracy,” “Sensitivity and Specificity,” “Area Under Curve,” “Sensitivity,” and “Specificity.” A full search strategy for each database is available in Supplementary material. All records were imported to Covidence, and duplicates were removed.

### Screening and data extraction

Two authors (AS and FR) independently screened titles and abstracts to assess for potential eligibility. Full texts of all screened studies were also reviewed by two authors for final selection. We manually searched bibliographies and citations of included studies and relevant systematic reviews to identify any additional relevant articles that might have been missed in the initial search. Eligibility assessment was done by two reviewers at all stages independently, and disagreements were resolved by involving a third reviewer (JKD, OP).

Two authors (AS and UJ) independently extracted information to a pre-formed data extraction sheet on Excel. Data obtained included information about the study (first author, year of publication, journal, study title, country, income region of the country according to the World Bank, aim of the study, study design, study setting, sample size (including size of training and test set), method of population selection, patient characteristics (age range, type of GI luminal pathologies), the AI algorithm used, the reference standard, the type of data used for analysis (per-image, per-lesion, or per-patient analysis), reported performance metrics (sensitivity, specificity, and area under the curve), validation of the model (internal or external) and sub-group data if present.

### Risk of bias assessment

The risk of bias was assessed by two authors (AS, UJ) independently using quality assessment for diagnostic accuracy studies-2 (QUADAS-2) tool (20). Domains for risk of bias included patient selection, index test, reference standard, and flow and timing, with the first three domains also considered in terms of applicability concerns. If one of the questions within the domain was scored at high risk of bias, the domain was scored as high risk. Disagreements in data extraction and quality assessment were resolved by discussion with a third reviewer (ZH or JKD).

### Data analysis

Where possible, we extracted 2 × 2 contingency tables or data to construct such tables. Contingency tables consisted of true-positive, false-positive, true-negative, and false-negative results, and were used to calculate sensitivity, specificity, and accuracy. External validation is important to establish the quality and generalizability of machine learning models (21), while internally validation alone has a potential to overestimate the accuracy of the model (22), therefore to estimate the accuracy of AI algorithms, we conducted a meta-analysis of studies that provided data for contingency tables separate for externally validated data (test data) and internally validated data (training data). If a study tested more than one AI model or more than one dataset, all contingency tables were included in the meta-analysis.

For all included studies, we entered the data provided into Review Manager (RevMan 5.4.1) software (23) where the sensitivity, specificity and their 95% confidence intervals (CIs) were presented in the form of forest plots and receiver operating characteristic (ROC) curves. This analysis utilized the sensitivity and specificity results from each included study using the metandi command for bivariate model in STATA version 17 (24) to generate hierarchical Summary ROC (HSROC) curves.

**FIGURE 1:**
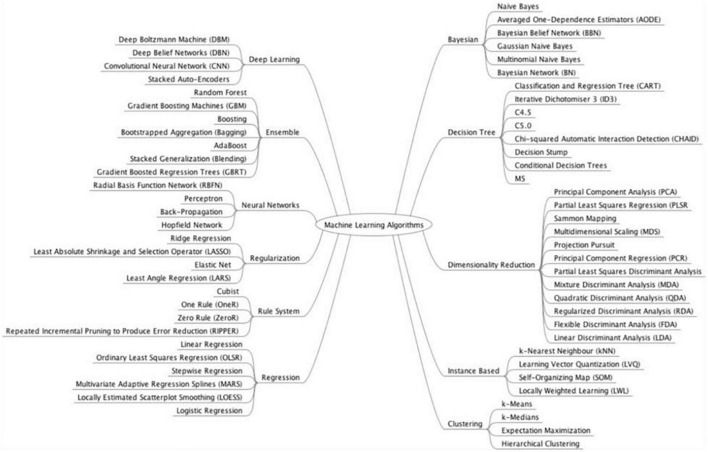
Model classification. Source: Brownlee (104).

Subgroup analyses were conducted based on the type of GI luminal pathologies, AI model, reference standard, and data used for analysis (image, lesions, or patients). Subgroup analyses were performed if at least four studies in each sub-group could be analyzed together. Subgroup analysis was conducted on the various GI luminal pathologies listed, types of AI models used, reference standards and types of input data (per patient, image, or lesion). We grouped AI models together according to their class (Figure 1). We performed separate analysis for externally and internally validated studies. We also conducted an exploratory analysis on internally validated studies to evaluate the diagnostic accuracy of AI models on internally validated data.

## Results

**FIGURE 2:**
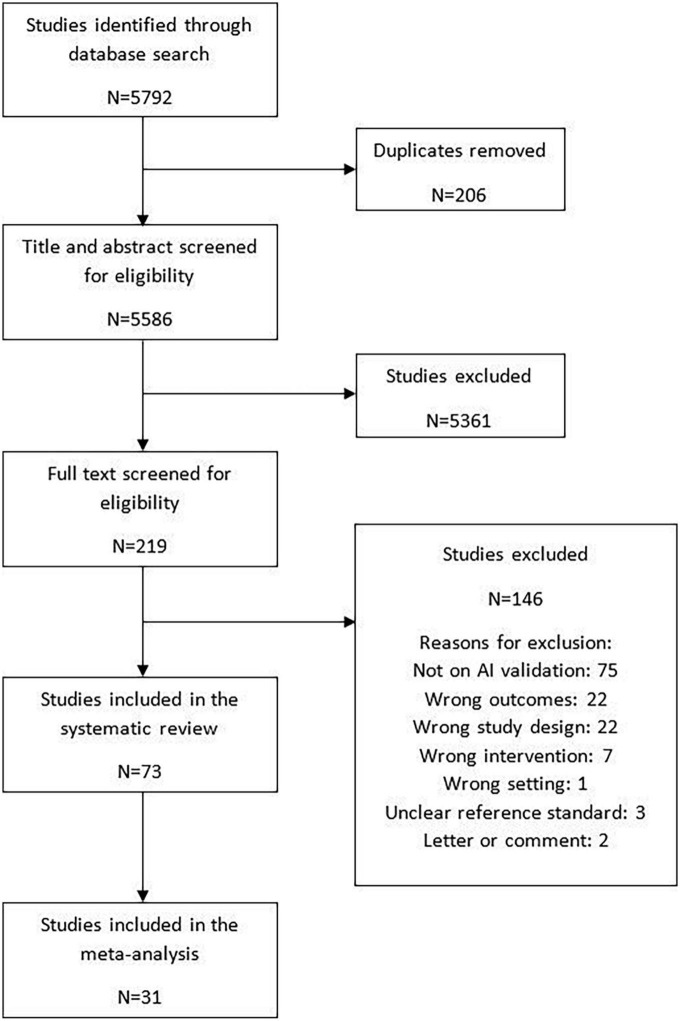
Study selection.

The search strategy identified 5,586 articles for title/abstract screening, of which 219 full texts were screened for eligibility. Altogether, 73 studies were included in the review. Of these studies, 68 studies were externally validated and five were internally validated. Among the externally validated studies 31 (42.5%) studies were included in the meta-analysis (10, 17, 25–53) while 37 (50.7%) studies were narratively synthesized due to insufficient information to calculate the contingency tables (11–14, 54–86). The five (6.8%) internally validated studies provided sufficient information to calculate contingency tables and were included in the exploratory analysis (87–91). The details of the study flow diagram have been shown in Figure 2.

Among the 73 included studies, 53 (72.6%) were case-control, 17 (23.3%) were cohort, one (1.4%) was a mixed-method study, and two (2.7%) studies failed to define the study design (Tables 1,2). Majority of the included studies were conducted in high income countries (HICs) (*n* = 54, 73.9%) and upper–middle income countries (UMICs) (*n* = 14, 19.2%), while four (5.5%) were conducted in both HICs and UMICs, and one (1.4%) in both HIC and LMIC. Diagnostic performance of AI was most tested on colorectal polyps (*n* = 29, 39.7%), followed by ulcers (*n* = 7, 9.6%), celiac disease (*n* = 6, 8.2%), IBD (*n* = 5, 6.8%), Barrett’s esophagus (*n* = 3, 4.1%), and gastric polyps (*n* = 3, 4.1%). Sixteen studies used AI to differentiate between benign and precancerous or cancerous polyps, while 10 studies used AI to diagnose more than one type of disease.

**TABLE 1:** Table of included studies (polyps).

Author and year published	Country and income region	Age	Study Design	AI model	Type of gastrointestinal pathologies	Reference standard	Sample size	External validation	Sensitivity	Specificity
Bagheri et al., 2019 (54)	Iran, UMIC	NR	Case-control	CNN	Polyps	Already labeled open dataset	358 images	Yes	0.83	0.99
Becq et al., 2019 (55)	USA, HIC	>50 years	Cohort	CNN	Polyps	Experts	50 videos	NR	0.99	NR
Blanes-Vidal et al., 2019 (56)	Denmark, HIC	NR	Cohort	CNN	Polyps	Experts	255 patients, 375 lesions, 11,300 images	Yes	0.97	0.93
Byrne et al., 2019 (25)	Canada, HIC	NR	Case-control	CNN	Adenomatous polyps	Histology	125 videos	Yes	0.83	0.98
Chen et al., 2018 (26)	Taiwan, HIC	>18 years	Case-control	CNN	Neoplastic polyps	Histology	2441 images	Yes	0.96	0.78
Cho et al., 2018 (59)	South Korea, HIC	19-75 years	Cohort	SVM	Polyps	Experts	113 patients	No	0.82	0.89
Ding et al., 2019 (28)	China, UMIC	NR	NR	CNN	Inflammation, ulcers, polyps, protruding lesions, vascular pathologies, bleeding, parasites, diverticula	Experts	8940 patients, 113,426,569 images	Yes	0.1	0.1
Fernandez- Esparrach et al., 2016 (60)	Spain, HIC	NR	Case-control	WM-DOVA energy maps	Polyps	Experts	612 images, 24 videos, 31 lesions	NR	0.70	0.72
Figueiredo et al., 2019 (29)	Portugal, HIC	Mean: 57 years	Case-control	SVM	Polyps	Histology	42 patients, 3040 images	Yes	LBP: 0.1	LBP: 0.78
									LBP (without inpainting): 0.1	LBP (without inpainting): 0.73
									LBP+P: 0.1	LBP+P: 0.8
									M-LBP: 0.97	M-LBP: 0.85
									M-LBP (without inpainting):	M-LBP (without inpainting):
									0.98	0.75
Fu et al., 2014 (86)	Taiwan, HIC	32-88 years	Case-control	SVM	Adenomatous polyps	Experts	100 patients, 100 images	Yes	NR	NR
Ganz et al., 2012 (61)	UK, HIC	NR	Case-control	gPb-OWT-UCM	Adenomatous polyps	Dataset 1: Experts Dataset 2: NR	Dataset 1: 52 images Dataset 2:93 images	Yes	NR	NR
Gross et al., 2011 (87)	Germany, HIC	NR	Cohort	SVM	Neoplastic polyps	Histology	214 patients, 415 lesions	No	0.95	0.90
Guo et al., 2020 (30)	China, UMIC	NR	Case-control	CNN	Polyps, erosions, ulcers, varices, advanced cancer	Experts	117,005 lesions, 327,121 images	Yes	Without annotation: 0.71 With annotation: 0.88	Without annotation: 0.71 With annotation: 0.90
Itoh et al., 2019 (62)	Japan, HIC	NR	Case-control	CNN	Polyps	Experts	951 patients, 1027 videos	Yes	0.86	0.97
Jin et al., 2020 (32)	Korea, HIC	NR	Cohort	CNN	Adenomatous polyps	Histology	2450 images	Yes	0.83	0.92
Kudo et al., 2020 (33)	Japan, HIC	Mean: 66.3 years	Cohort	SVM	Neoplastic polyps	Histology	100 lesions	Yes	0.97	0.1
Lee et al., 2020 (34)	Korea, HIC	NR	NR	DL	Polyps	Datasets 1 and 2: NR Dataset 3: Histology Dataset 4: Already labeled open dataset Datasets 5 and 6: Experts	361,567 images	Yes	0.89	0.87
Maslekar et al., 2010 (35)	UK, HIC	>18 years	Cohort	ANN	Lower GI pathologies: polyps, colitis, and colorectal cancer	Experts	350 patients	Yes	0.88	0.92
Mori et al., 2018 (65)	Japan, HIC	>18 years	Cohort	SVM	Neoplastic polyps	Experts	791 patients	NR	NR	NR
Ozawa et al., 2020 (69)	Japan, HIC	NR	Case-control	CNN	Polyps	Experts	12,895 patients, 16,418 images	Yes	0.92	NR
Patel et al., 2020 (37)	USA and China, HIC and UMIC	NR	Case-control	CNN	Adenomatous polyps	Experts annotated the open datasets	157 videos, 35,981 frames	Yes	VGG-19 (set 1): 0.84	VGG-19 (set 1): 0.63
									VGG-19 (set 2): 0.84	VGG-19 (set 2): 0.74
									VGG-19 BN (set 1): 0.72 VGG-19 BN (set 2): 0.79 ResNet50 (set 1): 0.81 ResNet50 (set 2): 0.71 DenseNet (set 1): 0.78 DenseNet (set 2): 0.71 SENet (set 1): 0.77 SENet (set 2): 0.81	VGG-19 BN (set 1): 0.79 VGG-19 BN (set 2): 0.74 ResNet50 (set 1): 0.67 ResNet50 (set 2): 0.71 DenseNet (set 1): 0.70 DenseNet (set 2): 0.71 SENet (set 1): 0.72 SENet (set 2): 0.62
									MnasNet (set 1): 0.77 MnasNet (set 2): 0.73	MnasNet (set 1): 0.66 MnasNet (set 2): 0.68
Poon et al., 2020 (38)	Hong Kong, HIC	NR	Dataset 1–4: case-control Dataset 6: cohort	CNN	Polyps	Datasets 1–4: already labeled open datasets Dataset 5: NR Dataset 6: Histology	4,443,728 images	Yes	Dataset A: 0.72 Dataset B: 0.72	Dataset A: 0.73 Dataset B: 0.92
Pu et al., 2020 (70)	Australia and Japan, HIC	Dataset 1: NR Dataset 2: >18	Case-control	CNN	Polyps	Histology	283 lesions, 1304 images	Dataset 1: No Dataset 2: Yes	NR	NR
Qadir et al., 2020 (71)	Norway, HIC	NR	Case-control	CNN, SSD	Polyps	Already labeled open dataset	69 videos	Yes	FP model of Faster R-CNN: 0.76 FP model of SSD: 0.57	FP model of Faster R-CNN: 0.1 FP model of SSD: 0.98
Renner et al., 2018 (39)	Germany, HIC	>18 years	Case-control	Deep NN	Adenomatous polyp	Dataset 1: already labeled open dataset Dataset 2: Histology	1079 images	Yes	0.92	0.62
Rodriguez-Diaz et al., 2020 (40)	USA, HIC	NR	Cohort	DL	Neoplastic polyps	Histology	405 patients, 887 lesions, 1265 images	Yes	0.96	0.84
Saito et al., 2020 (41)	Japan, HIC	Mean 60.1 years	Case-control	CNN	Polyps, nodules, epithelial tumors, submucosal tumors, and venous structures	Experts	385 patients, 48,091 images	Yes	0.91	0.8
Shi et al., 2019 (73)	China, UMIC	40-64 years	Cohort	SSD	Gastric polyps	Experts	43 patients	Yes	NR	NR
Shin et al., 2018 (43)	Norway, HIC	NR	Case-control	Dictionary based learning scheme, SVM	Polyps	Already labeled open dataset	1891 images	Yes	0.96	0.96
Shin et al., 2017 (42)	Norway, HIC	NR	Case-control	SVM, CNN	Polyps	Already labeled open dataset	1891 images	Yes	HOG+SVM: 0.7 Combined feature+SVM: 0.87 CNN (gray): 0.87 CNN (RGB): 0.91	HOG+SVM: 0.82 Combined feature+SVM: 0.81 CNN (gray): 0.32 CNN (RGB): 0.92
Silva et al., 2013 (44)	France, HIC	NR	Case-control	LVQ, AdaBoost, Hough transform	Polyps	Experts	1500 images	Yes	Hough transform: 0.94 Log Gabor: 0.42 Real Adaboost: 0.77 Attentional: 0.91 LVQ classification: 0.92	Hough transform: 0.15 Log Gabor: 0.89 Real Adaboost: 0.93 Attentional: 0.95 LVQ classification: 0.86
Song et al., 2020 (45)	Korea, HIC	NR	Cohort	CNN	Neoplastic polyps	Dataset 1: Histology Dataset 2: Experts	1169 images	Yes	Serrated polyps (set 1): 0.82 Serrated polyps (set 2): 0.74 MSMC (set 1): 0.84 MSMC (set 2): 0.88 DSMC (set 1): 0.59 DSMC (set 2): 0.62	Serrated polyps (set 1): 0.94 Serrated polyps (set 2): 0.94 MSMC (set 1): 0.75 MSMC (set 2): 0.72 DSMC (set 1): 0.93 DSMC (set 2): 0.97
Tajbakhsh et al., 2016 (47)	USA, HIC	NR	Case-control	RF	Polyps	Already labeled open dataset	300 videos	Yes	0.48	0.9
Tajbakhsh et al., 2015 (46)	USA, HIC	NR	Case-control	CNN	Polyps	Experts	40 videos	Yes	0.5	0.1
Taunk et al., 2019 (48)	USA, HIC	52-82 years	Cohort	SVM	Neoplastic polyps	Histology	26 patients, 47 lesions, 189 images	Yes	0.95	0.94
Tischendosrf et al., 2010 (74)	Germany, HIC	NR	Cohort	Linear classifiers, KNN, SVM	Neoplastic polyps	Histology	128 patients, 209 lesions	No	0.9	0.7
Urban et al., 2018 (14)	USA, HIC	NR	Cohort	CNN	Polyps	Experts	Dataset 1: 8641 images Datasets 2 and 3: 20 videos	Yes	0.93	0.93
Viscaino et al2019 (50)	Chile, HIC	NR	Case-control	SVM, DT, KNN, RF	Polyps	Already labeled open dataset	1132 images	Yes	SVM: 0.99 DT: 0.95 KNN: 0.98 RF: 0.97	SVM: 0.97 DT: 0.94 KNN: 0.97 RF: 0.95
Wang et al., 2018 (76)	China, UMIC	NR	Case-control	DL	Polyps	Dataset 1: expert Dataset 2 and 4: histology Dataset 3: already labeled open dataset Dataset 5: NR	Dataset 1: 5545 images, 1293 patients Dataset 2: 27,113 images, 1138 patients Dataset 3: 612 images Dataset 4: 138 videos, 110 patients Dataset 5: 54 videos, 54 patients	Yes	0.94	0.96
Wang et al., 2020 (52)	China, UMIC	0-18 years	Case-control	CNN	Polyps	Experts	1600 children, 41500 images	Yes	VGG-16 GAP (CP CHILD A): 0.96 VGG-19 GAP (CP CHILD A): 0.97 ResNet 101 GAP (CP CHILD A): 0.97 ResNet 152 GAP (CP CHILD A): 0.97 VGG-16 GAP (CP CHILD B) :0.97 VGG-19 GAP (CP CHILD B): 0.98 ResNet 101 GAP (CP CHILD B): 0.98 ResNet 152 GAP (CP CHILD B): 0.98	VGG-16 GAP (CP CHILD A): 0.99 VGG-19 GAP (CP CHILD A): 0.1 ResNet 101 GAP (CP CHILD A): 0.1 ResNet 152 GAP (CP CHILD A) : 0.1 VGG-16 GAP (CP CHILD B): 0.1 VGG-19 GAP (CP CHILD B): 0.1 ResNet 101 GAP (CP CHILD B) : 0.1 ResNet 152 GAP (CP CHILD B): 0.1
Xia et al., 2021 (53)	China, UMIC	NR	Case-control	CNN	Gastric erosions, polyps, ulcers, submucosal tumors, and xanthomas	Experts	797 patients, 1,023,955 images	Yes	0.96	0.1
Yang et al., 2020 (91)	China, UMIC	NR	Cohort	NVLLC, SVM	Polyps	Experts	1000 images	No	0.96	0.96
Yuan et al., 2017 (79)	Hong Kong, HIC	NR	Case-control	Stacked SAE	Polyps	Experts	4000 images	NR	NR	NR
Zachariah et al., 2020 (80)	USA, HIC	NR	Case-control	CNN	Adenomatous polyps	Histology	5912 images	Yes	NBI: 0.96 WL: 0.95 NBI+WL: 0.96 Diagnose and leave: 0.91	NBI: 0.90 WL: 0.88 NBI+WL: 0.90 Diagnose and leave: 0.88
Zhang et al., 2017 (81)	Hong Kong, HIC	NR	Case-control	CNN	Adenomatous polyps	Dataset 1: histology Dataset 2: already labeled open database	2262 images	No	NR	NR
Zhang et al., 2019 (82)	China, UMIC	NR	Case-control	SSD, CNN	Gastric polyps	Experts	575 images	Yes	NR	NR
Zhao et al., 2011 (83)	Hong Kong, HIC	NR	Case-control	HMM, KNN	Polyps	Experts	1520 images	NR	NR	NR
Zhao et al., 2015 (84)	USA, HIC	NR	Case-control	HMM	Polyps	Experts	5029 images	No	NR	NR

ANN: artificial neural network; CNN: convolutional neural network; DL: deep learning; DSMC: deep submucosal cancer; DT: decision trees; GAP: global average pooling; gPb-OWT-UCM: global probability of boundary followed by the oriented watershed transform and ultrametric contour maps; FP: false positive; HIC: high-income country; HMM: hidden markov model; HOG: histogram of oriented gradient; KNN: k-nearest neighbor; LBP: local binary pattern; LMIC: lower-middle-income country; LVQ: learning vector quantization; M-LBP: monogenic local binary pattern; MSMC: mucosal or superficial submucosal tumor; NBI: narrow band imaging; NVLLC: normal variant locality-constrained linear coding; R-CNN: region-based convolutional neural network; RF: random forest; RGB: red, green, blue; SAE: sparse autoencoder; SENet: squeeze-and-excitation network; SSD: single shot detector; SVM: support vector machine; UMIC: upper-middle income country; WL: white light; WM-DOVA: window median depth of valleys accumulation; NR: not reported.

**TABLE 2:** Table of included studies (other gastrointestinal luminal pathologies).

Author and year published	Country and income region	Age	Study design	AI model	Type of gastrointestinal pathologies	Reference standard	Sample size	External validation	Sensitivity	Specificity
Charisis et al. (57)	Greece, HIC	NR	Case-control	BEEMD	Ulcers	Experts	6 patients, 80 images	No	0.95	0.96
Charisis and Hadjileontiadis (58)	Greece, HIC	NR	Case-control	SVM	Crohn’s disease	Dataset 1: experts Dataset 2: already labeled open dataset	Dataset 1: 13 patients, 800 dataset 2: 102 images	No	NR	NR
de Groof et al. (27)	Netherlands, HIC	NR	Case-control	CNN	Barrett’s neoplasia	Experts	20 patients	Yes	0.76	0.86
Huang et al. (88)	Taiwan, HIC	NR	Case-control	HHDF-SVM	GERD	Experts	147 patients	No	0.95	0.93
Hwang et al. (31)	South Korea, HIC	NR	Case-control	CNN	Hemorrhagic and ulcerative lesions	Experts	13,316 images	Yes	Binary model: 0.95 Combined model: 0.98	Binary model: 0.98 Combined model: 0.96
Klang et al. (63)	Israel, HIC	21–40 years	Case-control	CNN	Crohn’s disease	Histology and	49 patients,	No	NR	NR
						expert	17,460 images			
Li et al. (64)	China and USA, UMIC and HIC	NR		SVM	Celiac disease	Experts	23 patients, 460 images	Yes	NR	NR
Maeda et al. (10)	Japan, HIC	Mean: 50 years	Cohort	SVM	Ulcerative colitis	Histology	187 patients, 22,835 images	Yes	0.74	0.97
Mossotto et al. (11)	UK, HIC	1.6–17.6 years	Case-control	SVM	Ulcerative colitis and Crohn’s disease	Experts	287 patients	Yes	NR	NR
Namikawa et al. (36)	Japan, HIC	NR	Case-control	CNN	Gastric cancer	Experts	95,721 images	Yes	0.99	0.93
Otani et al. (66)	Japan, HIC	Mean: 63.3 years	Case-control	Deep neural network, SSD	Erosions, ulcers, angioectasias, and tumors	Experts	455 patients,	No	NR	NR
Owais et al. (67)	Korea, HIC	NR	Case-control	CNN, RNN	All GI pathologies	Already labeled open dataset	52,471 images, 77 videos	Yes	NR	NR
Owais et al. (68)	Korea, HIC	NR	Case-control	CNN, RNN	All GI pathologies	Already labeled open dataset	52,471 images, 77 videos	Yes	NR	NR
Ozawa et al. (12)	Japan, HIC	14–83 years	Case-control	CNN	Ulcerative colitis	Experts	558 patients,	Yes	NR	NR
							30,285 images			
Sevo et al. (72)	Bosnia, UMIC	NR	Case-control	Kernel based edge detection	Inflammation	Experts	3 videos	NR	NR	NR
Struyvenberg et al. (89)	Sweden and Netherlands, HIC	NR	Case-control	CNN	Barrett’s neoplasia	Dataset 1: Experts Datasets 2 and 3: Histology	1,587 videos	No	Dataset 3: 0.88 Dataset 4: 0.85	Dataset 3: 0.77 Dataset 4: 0.83
Swager et al. (90)	Netherlands, HIC	Mean: 67 years	Case-control	SVM, DA, AdaBoost, RF, k-NN, naive Bayes, linear regression, and logistic regression	Barrett’s neoplasia	Histology	60 images	No	0.9	0.93
Syed et al. (13)	Pakistan, Zambia, and USA, LMIC and HIC	Median: 31 months	Case-control	CNN	Environmental enteropathy and celiac disease	Histology and clinical findings	102 patients, 3,118 images	No	NR	NR
Tenorio et al. (49)	Brazil, UMIC	NR	Cohort	DT, Bayesian inference, KNN, SVM, ANN	Celiac disease	Histology	216 patients	Yes	0.93	0.96
Vecsei et al. (75)	Austria, HIC	NR	Case-control	KNN, SVMs, and Bayes classifier	Celiac disease	Histology	391 images	No	NR	NR
Wang et al. (77)	China, UMIC	NR	Case-control	CNN	Ulcers	Experts	1,416 videos, 1,416 patients	No	0.92	0.92
Wang et al. (51)	China, UMIC	NR	Case-control	CNN	Ulcers	Experts	47,202 images	Yes	0.9	0.9
Wang et al. (78)	China and USA, UMIC and HIC	NR	Case-control	DL, SVM, KNN, LDA, CNN	Celiac disease	Experts	25 patients, 2,140 images	No	0.89	0.9
Zheng et al. (85)	China, UMIC	Mean: 48.5 years	Case-control	CNN	H. pylori infection	HHistology and breath test	1959 patients, 15,484 images	Yes	0.81	0.9
Zhou et al. (17)	China, Hong Kong, and USA, UMIC and HIC	≥ *18* years	Case-control	CNN	Celiac disease	Experts	21 patients	Yes	0.1	0.1

ANN, artificial neural network; BEEMD, bidimensional ensemble empirical mode decomposition; CNN, convolutional neural network; DA, discriminant analysis; DL, deep learning;; GERD, Gastro-esophageal reflux disease; HHDF-SVM, hierarchical heterogeneous descriptor fusion support vector machine; HIC, high-income country; KNN, k-nearest neighbor; LDA, linear discriminant analysis; LMIC, lower-middle-income country;, re RF, random forest; RNN, recurrent neural network; SSD, single shot detector; SVM, support vector machine; UMIC, upper-middle income country; NR, not reported.

Only 23 (31.5%) studies reported the age of the participants. Nineteen studies (82.6%) reported on adult population, three (13%) on pediatric population and one (4.3%) study reported on both. At least one dataset used in 16 (21.9%) studies, was a publicly available database (34, 37–39, 42, 43, 47, 50, 54, 58, 60, 67, 68, 71, 76, 81). Sixty three (86.3%) studies used their own data (10–14, 17, 25–36, 38–41, 44–46, 48, 49, 51–53, 55–59, 61–66, 69, 70, 72–91), while 18 (28.6%) collected data prospectively (11, 13, 26, 27, 32, 35, 38, 39, 45, 55, 56, 59, 65, 70, 73, 74, 80, 87).

### Methodological quality of the included studies

**FIGURE 3:**
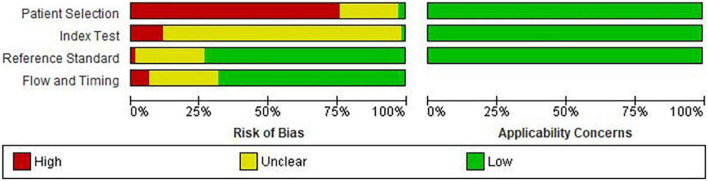
Risk of bias in the included studies.

Details of risk of bias and applicability concerns are presented in Figure 3. Fifty-six studies (76.7%) had a high risk of bias in patient selection (11–13, 17, 25–27, 29–31, 34, 36–39, 41–44, 46, 47, 50–54, 57, 58, 60–72, 74–79, 81, 83–86, 88–91), mostly due to the case-control study design (*n* = 54, 74%) (11–13, 17, 25–27, 29–31, 34, 36–39, 41–44, 46, 47, 50–54, 57, 58, 60–64, 66–72, 75–79, 81, 83–86, 88–91). Two studies had low risk of bias for patient selection because of random participant sampling, and due to appropriate exclusion of participants (55, 87), while 15 studies (20.5%) did not provide sufficient information for risk classification (10, 14, 28, 32, 33, 35, 40, 45, 48, 49, 56, 59, 73, 80, 82). Majority of the studies had unclear (*n* = 63, 86.3%) (10, 11, 13, 14, 17, 25–33, 35, 36, 38, 39, 41–45, 48–55, 57–59, 61–88, 91) or high (*n* = 9, 12.3%) (12, 34, 37, 46, 47, 56, 60, 89, 90) risk of bias for index test, which was most often due to insufficient information on blinding of the index test (*n* = 67, 91.8%) (11–14, 17, 25–33, 36–39, 41–64, 66–72, 74–91) and pre-specification of diagnostic threshold (*n* = 61, 83.6%) (10, 11, 13, 14, 17, 25, 26, 29–33, 35, 36, 38, 39, 41–45, 48–55, 57–59, 61–88, 91). Fifty-four (74.0%) studies were classified as low risk for reference standard due to correct classification of the condition and interpretation of diagnostic test without the knowledge of the index test (10–13, 17, 26–36, 38, 39, 41, 44, 45, 47–49, 51–53, 56, 58–63, 69–72, 74–79, 81–90), while 49 (67.1%) were categorized as low risk for flow and timing biases due to inclusion of all the participants, appropriate interval between index test and reference standard and due to provision of same reference standard to the all the ails of risk of bias and applicab participants (10, 11, 13, 17, 26–33, 35–37, 41, 45, 48, 51–53, 55–64, 69, 70, 72, 74–79, 81–85, 87–89, 91). No study had concerns about applicability in all three domains.

### Detection of any gastrointestinal luminal pathology by externally validated artificial intelligence models

**FIGURE 4:**
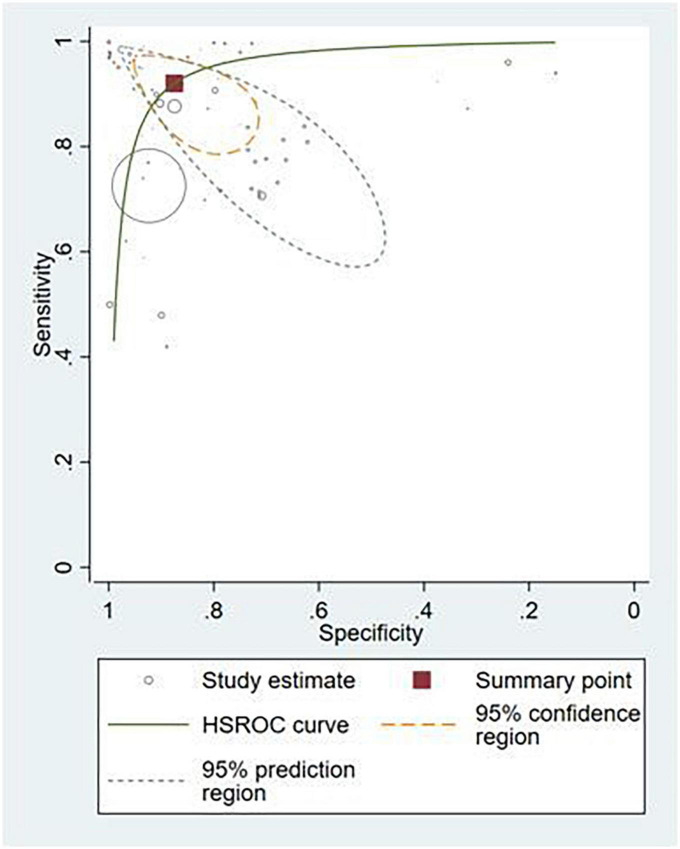
Hierarchical summary ROC curve for externally validated studies.

Thirty-six studies provided sufficient information to calculate contingency table values (10, 17, 25–53, 87–91). Of these, 31 studies (86.1%) conducted external validation and were included in the meta-analysis (10, 17, 25–53). Contingency tables of per-image analyses were used for the meta-analysis; if the study did not report per-image analyses, then per-lesion results were included. If neither per-image nor per-lesion analyses were reported, per-patient results were used. The hierarchical summary ROC curve of these studies is shown in Figure 4. The overall sensitivity from these studies was 91.9% (95% CI: 89.0–94.1) while the specificity was 91.7% (95% CI: 87.4–94.7).

### Subgroup analyses based on different gastrointestinal luminal pathologies

**FIGURE 5:**
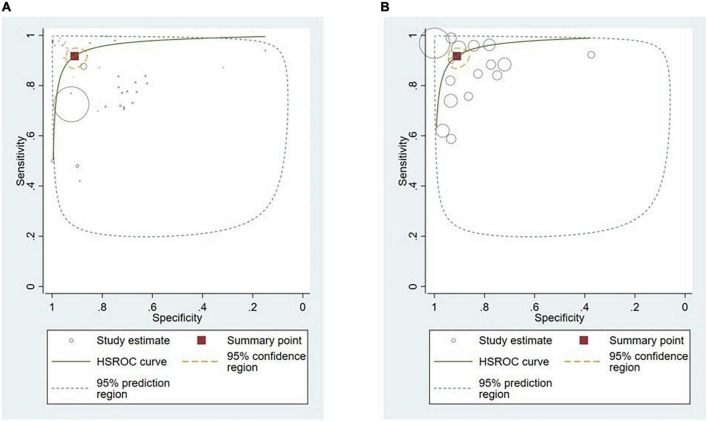
Hierarchical summary ROC curve for detecting GI luminal diseases. **(A)** GI polyps and **(B)** GI neoplasms.

Polyps (*n* = 13, 36.1%) (29, 34, 38, 42–48, 50, 52, 91) and neoplasms (*n* = 14, 38.9%) (25–27, 32, 33, 36, 37, 39, 40, 45, 48, 87, 89, 90) were the most commonly reported GI luminal pathologies. Two studies (5.5%) modeled AI for the detection of celiac disease (17, 49), while ulcers (51), IBD (10), and GERD (88) were reported in one study (2.8%) each. The sensitivity of AI models for detecting polyps was 94.0% (95% CI: 88.7–97.0) and the specificity was 95.4% (89.9–98.0) (Figure 5A). AI models demonstrated a sensitivity of 86.3% (95% CI: 82.0–89.7) and specificity of 82.6% (95% CI: 75.6–87.9) for diagnosing GI luminal neoplasms (Figure 5B). No subgroup analyses were performed for the other pathologies due to insufficient number of studies.

### Subgroup analyses based on artificial intelligence model

Twenty-two studies used deep learning (61.1%) (17, 25, 26, 28, 30–32, 34, 36–42, 44–46, 51–53, 89) and 15 studies used ensemble methods (41.7%) (10, 27, 29, 33, 42–44, 47–50, 87, 88, 90, 91) for the detection of GI luminal pathologies, while the other AI models reported by the studies included artificial neural networks (ANN) (*n* = 1, 2.8%) (35), k-nearest neighbors (kNN) (*n* = 1, 2.8%) (50) learning vector quantization (LVQ) (*n* = 1, 2.8%) (44) and decision trees (*n* = 1, 2.8%) (50). The sensitivity of deep learning models to detect any GI luminal pathology was 89.8% (95% CI: 85.9–92.7) while that of ensemble methods was 95.4% (95% CI: 91.3–97.6). The specificity of deep learning and ensemble methods was 91.9% (95% CI: 85.7–95.6) and 90.9% (95% CI: 86.2–94.1), respectively (Supplementary Figures). ANN, k-NN, LVQ, and decision trees weren’t included in the subgroup analyses due to the lack of sufficient number of studies.

### Subgroup analyses based on different reference standards

Twenty (55.5%) studies used expert diagnosis (17, 27, 28, 34, 36–38, 41–47, 50–53, 88, 91) and 14 (38.9%) studies used histopathology as the reference standard (10, 25, 26, 32, 33, 38–40, 45, 48, 49, 87, 89, 90). The sensitivity and specificity of AI models when compared to expert opinion was 90.5% (95% CI: 86.5–93.4) and 93.3% (95% CI: 88.1–96.4). Studies that used histopathology as the reference standard reported 79.8% (95% CI: 38.2–96.2) sensitivity and 97.6% specificity (95% CI: 94.0–99.1) (Supplementary Figures).

### Detection of any gastrointestinal luminal pathology by internally validated artificial intelligence models

The performance of AI models on internally validated data (*n* = 5, 13.8%) (87–91) was similar to its performance on externally validated data (sensitivity: 91.9%; 95% CI: 89.0–94.1, specificity: 91.7%; 95% CI: 87.4–94.7). The exploratory analysis of internally validated studies showed a sensitivity of 92.9% (95% CI: 89.3–95.4) and specificity of 90.1% (83.8–94.1%).

## Discussion

This meta-analysis aimed to synthesize the existing evidence regarding diagnostic accuracy of AI models in detecting common GI luminal pathologies as compared to the reference standard. AI models such as deep learning techniques and ensemble methods were the most commonly deployed models to detect GI luminal pathologies with reported high sensitivity and specificity (> 90%). The most common GI luminal pathologies investigated were the occurrence of polyps and GI neoplasm, both of which had high accuracy.

AI is rapidly gaining momentum across various industries including healthcare. AI has been used in diagnostics, management and improving administrative efficiency of healthcare systems (92). Benefits of AI have been reported in several fields of medicine including cardiology (93), radiology (94) and pediatrics (95). Gastroenterologists are a subset of clinicians who handle large volumes of clinical as well as imaging data obtained through various procedures such as endoscopy and colonoscopy (96). AI algorithms have proven to show high accuracy in diagnosing various gastroenterological pathologies such as polyps, Barrett’s esophagus, celiac disease and Inflammatory Bowel disease (96). These algorithms range from neural networks to ensemble methods such as support vector machines and deep learning techniques (96). In the current review, deep learning and ensemble methods were the most commonly used AI models in the included studies. Our study reported a sensitivity of 86.3% and specificity of 82.6% for diagnosing GI luminal neoplasms. This is similar to the meta-analysis done by Zhang et al. which reported a sensitivity of 94% and specificity of 82% for AI models on esophageal neoplasms only (97). Similarly, a meta-analysis of the accuracy of AI was superior to experts in the detection of conditions such as Barrett’s esophagus and helicobacter Pylori infection (98).

From the 73 studies included in this review, only one study was from a LMIC (Pakistan) while the remaining were from UMIC or HICs. As demonstrated by the Global Burden of Disease data, the burden of benign and neoplastic GI pathologies in LMICs is significantly higher than UMIC and HICs (99). The disproportionately high prevalence of disease alongside poor access to healthcare facilities, lack of equipment and trained professionals in these regions contributes to poor health outcomes (100). It is in these regions that AI may play a disruptive role in healthcare. The areas of impact of AI in LMICs have been well documented by the United States Agency for International Development (USAID) where physician decision support systems may be one of the domains where deployment of these algorithms could help increase access and high-quality care for medical conditions such as GI pathologies which are highly prevalent in these regions (101). Despite this potential implication and similar to other complex conditions prevalent in LMICs (102), published literature on use of AI in healthcare in these regions is lacking.

As seen in the current review as well, majority of literature on AI in medicine is retrospective. The number of prospective studies where AI is implemented in a clinical setting is limited. There is lack of methodological rigor in the design and conduct of the studies published with the risk of bias in most of the domains stated as unclear or high. It is for this reason that clinicians specifically and health systems in general, lack the confidence in such algorithms thus precluding implementation and large-scale benefits of AI in real world settings (103). Future work needs to include methodological rigor in prospectively designed collaborative studies to demonstrate the use of AI in detection of GI pathologies in HICs as well as LMICs. With higher accuracy of detecting these conditions as compared to a human interpreter, the implications of AI in terms of efficient use of resources and better patient outcomes can be substantial.

To the best of our knowledge, this is the first meta-analysis on the diagnostic accuracy of AI algorithms in both upper and lower GI pathologies. Compared to other reviews, we also explored various subgroups in terms of best performing AI models and across world regions where these studies had been conducted. However, there are some limitations of our work. Our review included studies published in the English Language only. This review focused on certain GI luminal pathologies and is no means an exhaustive exercise to include other GI conditions such as liver, pancreatic and biliary pathology. We also did not include studies where AI models utilized in gastroenterological radiology were compared to expert radiologists.

AI models have the potential to accurately diagnose GI lesions based on endoscopic findings as compared to experts. These results could have significant implications for patient related outcomes in resource constrained settings where trained personnel to interpret these images are limited. However, to fully reap the benefits of AI models, prospectively designed large, multi-center studies are required to demonstrate the effectiveness of the results and for implementation of AI into routine clinical practice across HIC and LMICs.

## Data availability statement

The original contributions presented in this study are included in the article/Supplementary material, further inquiries can be directed to the corresponding author.

## Author contributions

AS, UJ, and FR were involved in the literature search, data collection, analysis, and writing. AR were involved in data analysis, interpretation, and writing. OP, ZH, ZP, and JD were involved in study design, data analysis, interpretation, and writing of the manuscript. All authors contributed to the article and approved the submitted version.
