# Supplementary material for: Diagnostic accuracy of artificial intelligence for detecting gastrointestinal luminal pathologies: A systematic review and meta-analysis
Source: Front Med (Lausanne). 2022 Nov 4;9:1018937. doi: 10.3389/fmed.2022.1018937 (PMC9672666; doi:10.3389/fmed.2022.1018937)

Supplementary Material

Supplementary Table 1: Search strategy

“Algorithms”[mh] OR "Artificial Intelligence"[mh] OR "Machine Learning"[mh] OR "Deep Learning"[mh] OR “Supervised Machine Learning”[mh] OR “Unsupervised Machine Learning”[mh] OR “Algorithms”[tiab] OR "Artificial Intelligence"[tiab] OR "Machine Learning"[tiab] OR "Deep Learning"[tiab] OR “Supervised Machine Learning”[tiab] OR “Unsupervised Machine Learning”[tiab])<br><br>AND ("Gastroenterology"[mh] OR "Celiac Disease"[mh] OR "Inflammatory Bowel Diseases"[mh] OR "Irritable Bowel Syndrome"[mh] OR “polyps”[mh] OR “Crohn Disease”[mh] OR Gastro*[tiab] OR "Celiac Disease"[tiab] OR "Inflammatory Bowel Disease*"[tiab] OR "Irritable Bowel Syndrome"[tiab] OR “polyp*”[tiab] OR “Crohn Disease”[tiab])<br><br>AND (“Endoscopy”[mh] OR “scopy”[tiab] OR “Capsule endoscopy”[mh] OR “Endomicroscopy”[tiab] OR “Ultrasound manometry”[tiab] OR “Diagnosis”[mh] OR “Colonoscopy”[mh] OR “diagnos*”[tiab])<br><br>AND (“accuracy”[tiab] OR “Sensitivity and specificity”[mh] OR “Area under curve”[mh] OR “sensitivity”[tiab] OR “specificity”[tiab] OR “area under the curve”[tiab] OR “area under curve”[tiab])	PubMed strategy
(artificial intelligence OR machine learning OR deep learning OR neural network OR computer vision) AND (gastr* OR celiac disease OR inflammatory bowel disease OR polyps OR irritable bowel syndrome OR endoscopy OR capsule endoscopy OR endomicroscopy OR ultrasound manometry OR diagnosis OR colonoscopy OR expert) AND (diagnosis OR accuracy OR specificity OR sensitivity OR area under curve)	CINAHL
No search strategy attempted due to lack of articles	Cochrane

Supplementary Figure 1: Hierarchical summary ROC curve for AI models

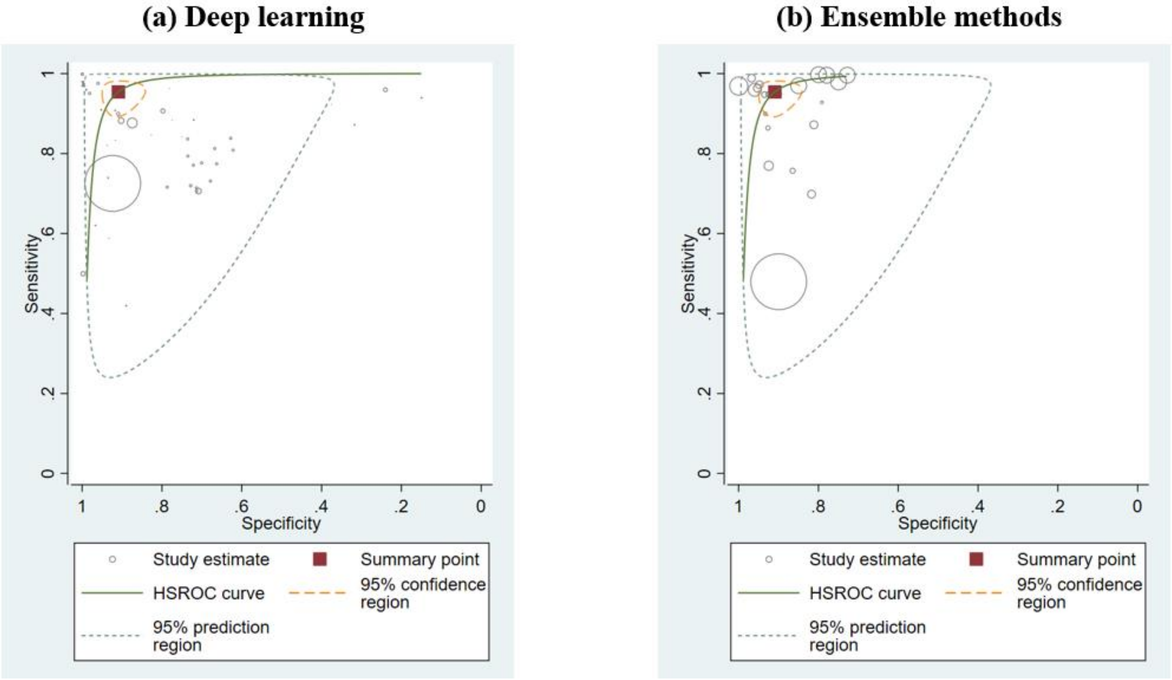

Supplementary Figure 2: Hierarchical summary ROC curve for reference standards

(a) Experts **(b) Histopathology**

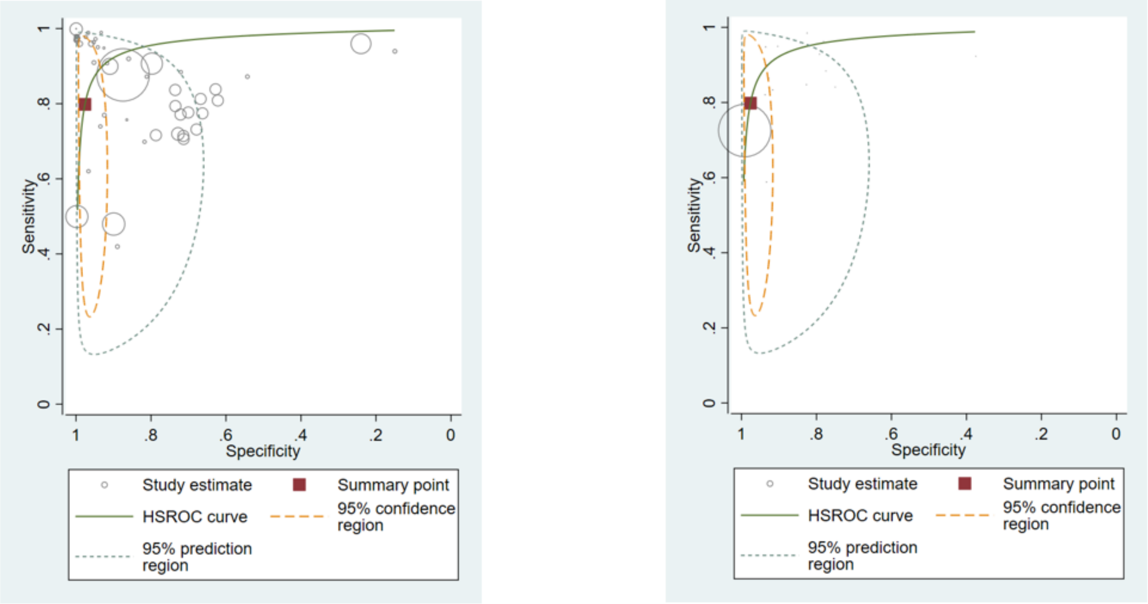

Supplementary Figure 3: Hierarchical summary ROC curve for internally validated models

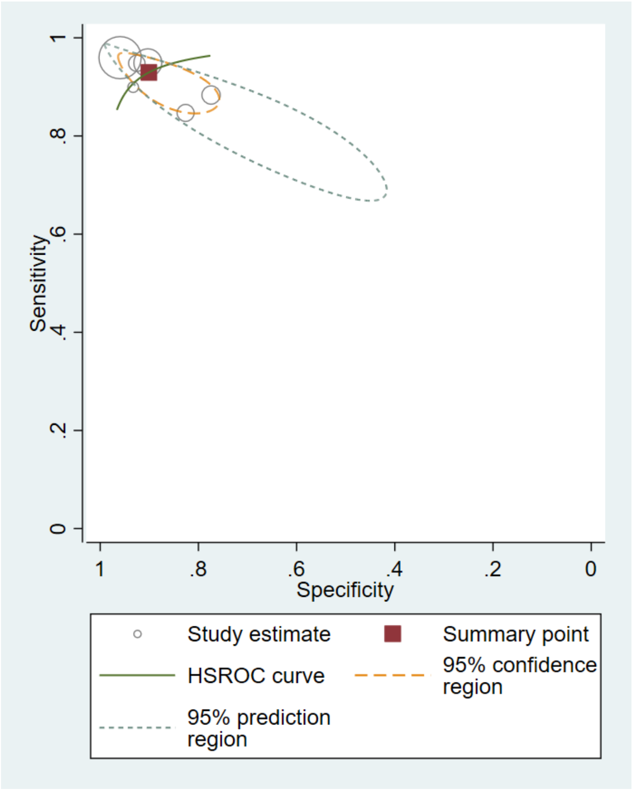

Supplement: Supplementary file 1 [file Data_Sheet_1.pdf]
